# Supplementary material for: Multiple Sclerosis: Enzymatic Cross Site-Specific Recognition and Hydrolysis of H3 Histone by IgGs against H3, H1, H2A, H2B, H4 Histones, Myelin Basic Protein, and DNA
Source: Biomedicines. 2022 Oct 21;10(10):2663. doi: 10.3390/biomedicines10102663 (PMC9599260; doi:10.3390/biomedicines10102663)
Supplement: Supplementary file 1 [file biomedicines-10-02663-s001.zip › biomedicines-1883270-supplementary.pdf]

Supplementary data

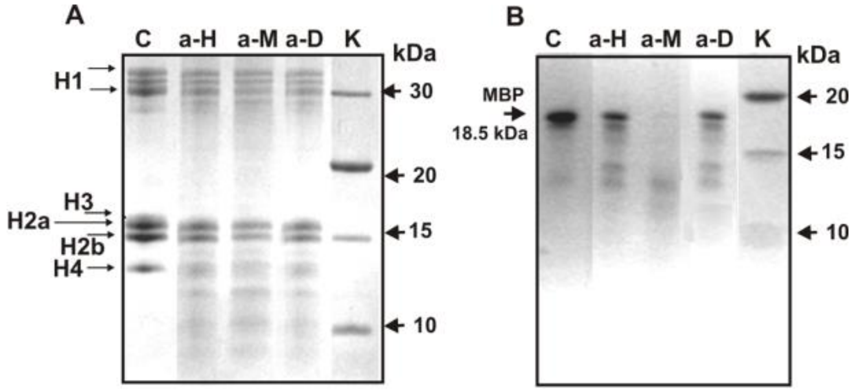

Supplementary Figure S1. SDS-PAGE analysis of five histones hydrolysis by IgGs (abzymes) against five histones (lane a-H), MBP (lane a-M), and DNA (lane-D) (A) as well as human MBP by IgGs against 5 histones (lane a-H; a mixture of 5 histones) and Abs against MBP (lane a-M), and DNA (lane-D) (B). C lanes correspond to five histones (A) and MBP (B) incubated in the absence of IgGs. Lanes K - proteins with known molecular masses (MMs) (A and B). MBP and a mixture of five histones with and without IgGs (0.03 mg/ml) were incubated for 11 h, while with anti-DNA Abs were for 24 h [46,47].

Figure S1A demonstrates effective hydrolysis of five histones by anti-histones, anti-MBP, and anti-DNA IgGs, while Supplementary Figure S1B hydrolysis of MBP by these IgGs.

Electrophoretically homogeneous human MBP preparations, unfortunately, were not available. In a consequence of cDNA alternative splicing as well as different MBP partial hydrolysis in the brain of different humans, MBP preparations could consist of several related forms (18.5, 17.5, and ≤14.0 kDa) and products of the protein hydrolysis [21,22]. Supplementary Figure S1B shows MBP splitting by IgGs against MBP, five histones, and DNA. Line C of Figure S1B shows the heterogeneity of starting MBP preparation containing mainly 18.5 kDa protein forms. After 11 and 24 h of the incubation with three IgG preparations, the relative content of all MBP forms decreased remarkably compared to control (lane C).

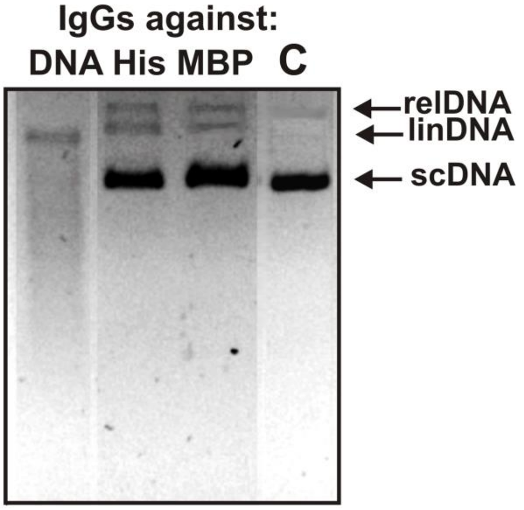

Supplementary Figure S2. Analysis of the relative activity of IgGs (10 μg/ml) against DNA, five different histones, and MBP in the hydrolysis of scDNA for 3 h. Designations of IgGs are shown in the Figure [46,47].

It can be seen that after 3 h of the incubation with IgGs against DNA, they completely hydrolyze scDNA to the linear form of DNA (two or more breaks per molecule) and oligonucleotides of different lengths. IgGs against five histones and MBP effectively hydrolyzed scDNA during this time by about 25-35% with the formation of a relaxed (one break per molecule) and linear form of DNA (2 or more breaks per molecule). An increase in the IgGs concentration against MBP and five histones and the incubation time results in the formation of short splitting products, as in the case of IgGs against DNA [46,47].

Supplementary Table S1. Several different characteristics of MS patients [31,32]

№	MS subtypes	Sex	Age (years)	Disease duration (years)	EDSS*
Debut of multiple sclerosis (DMS)**					
1	DMS1	F	26	0.083	3.0
2	DMS2	F	24	0.17	4.5
3	DMS3	F	21	0	2.0
4	DMS4	M	30	0	3.5
5	DMS5	M	29	0	3.5
6	DMS6	F	20	0.083	1.5
7	DMS7	F	26	0.083	2.0
8	DMS8	F	43	0	4.0
Average values	8 patients	6 F and 2 M	27.4 ±7.2	0.05±0.06	3.0±1.1
Remitting multiple sclerosis (RMS)					
9	RMS 2	M	46	16	4.0
10	RMS3	F	22	1	3.5
11	RMS4	F	53	23	3.5
12	RMS5	F	38	12	3.0
13	RMS6	F	58	20	3.0
14	RMS7	M	38	9	4.0
15	RMS8	F	45	11	4.0
17	RMS9	M	52	3	2.0

*Kurtzke's expanded disability status scale (EDSS)

**Debut of multiple sclerosis (DMS) corresponds to the first coming of patients in the clinic for research after the early manifestations of signs of this pathology.
